# A Parameter Estimation of Photovoltaic Models Using a Boosting Flower Pollination Algorithm

**DOI:** 10.3390/s23198324

**Published:** 2023-10-08

**Authors:** Shuai Liu, Yuqi Yang, Hui Qin, Guanjun Liu, Yuhua Qu, Shan Deng, Yuan Gao, Jiangqiao Li, Jun Guo

**Affiliations:** 1 School of Civil and Hydraulic Engineering, Huazhong University of Science and Technology, Wuhan 430074, Chinad202081167@hust.edu.cn (G.L.); d202280600@hust.edu.cn (Y.Q.);; 2 Hubei Key Laboratory of Digital Valley Science and Technology, Huazhong University of Science and Technology, Wuhan 430074, China; 3 Hubei Key Laboratory of Intelligent Yangtze and Hydroelectric Science, China Yangtze Power Co., Ltd., Yichang 443000, China

**Keywords:** photovoltaic models, parameter estimation, energy systems, flower pollination algorithm

## Abstract

An accurate and reliable estimation of photovoltaic models holds immense significance within the realm of energy systems. In pursuit of this objective, a Boosting Flower Pollination Algorithm (BFPA) was introduced to facilitate the robust identification of photovoltaic model parameters and enhance the conversion efficiency of solar energy into electrical energy. The incorporation of a Gaussian distribution within the BFPA serves the dual purpose of conserving computational resources and ensuring solution stability. A population clustering strategy is implemented to steer individuals in the direction of favorable population evolution. Moreover, adaptive boundary handling strategies are deployed to mitigate the adverse effects of multiple individuals clustering near problem boundaries. To demonstrate the reliability and effectiveness of the BFPA, it is initially employed to extract unknown parameters from well-established single-diode, double-diode, and photovoltaic module models. In rigorous benchmarking against eight control methods, statistical tests affirm the substantial superiority of the BFPA over these controls. Furthermore, the BFPA successfully extracts model parameters from three distinct commercial photovoltaic cells operating under varying temperatures and light irradiances. A meticulous statistical analysis of the data underscores a high degree of consistency between simulated data generated by the BFPA and observed data. These successful outcomes underscore the potential of the BFPA as a promising approach in the field of photovoltaic modeling, offering substantial enhancements in both accuracy and reliability.

## 1. Introduction

In recent years, with the exponential growth of the global population and the swift expansion of the economy, the consumption of large-scale traditional energy (such as oil, coal, natural gas, etc.) has had a negative impact on the environment beyond estimation [1]. For this reason, renewable energy has become an alternative to fossil energy and has attracted much attention in the world [2]. Solar energy is converted into photoelectricity through solar radiation [3]. Due to the popularity, harmlessness, and permanence of solar energy, solar energy has become one of the major renewable energy sources [4]. However, photovoltaic power generation devices are usually exposed to harsh outdoor environments and are highly susceptible to weather changes [5]. In order to ensure the high efficiency of photovoltaic conversion into electricity, more and more researchers have conducted in-depth research on the optimal parameters of the photovoltaic (PV) system [6,7,8,9,10].

Accurate modelling of photovoltaic systems by mathematical models to simulate the behavior of photovoltaic cells has achieved significant progress over the past decades. As an important semiconductor material [11,12], a diode is an important component of the photovoltaic cell/module model. Widely accepted photovoltaic cell models fit the observed current–voltage (*I*-*V*) data through an equivalent circuit composed of diodes, including single-diode models (SDM), double-diode models (DDM), and three-diode models (TDM) [13], which are the numbers of parameters that a model needs to estimate increases with the complexity of the equivalent circuit model. Simultaneously, the accurate identification of photovoltaic model parameters is not only beneficial to the performance evaluation of photovoltaic cells under concealed light and different temperatures but also has a non-negligible effect on battery design optimization and quality control [14]. Therefore, there is an urgent need for research on parameter estimation in photovoltaic cell systems.

PV model parameter identification methods can be mainly divided into two categories: deterministic optimization methods and heuristic algorithms. Deterministic optimization methods usually require the optimization problem to be convex or to ensure that the problem model can be differentiated, and the optimization results obtained usually have large deviations. A heuristic algorithm regards the optimization problem as a “black box” [15] and does not need to consider the specific form of the problem; it has been widely used for the parameter evaluation of PV systems. An improved queue search optimization algorithm (IQCODE) was developed by Abd El-Mageed et al. [16]; it is based on the differential evolution algorithm and uses random boundary patching technology to extract parameters for single and double diodes and photovoltaic modules. Kharchouf et al. [17] introduced the Lambert W function into the differential evolution algorithm, and a preliminary meta-heuristic technology was proposed to identify unknown parameters in the current–voltage characteristics of different PV models. Ali et al. [18] utilized an atomic orbital search algorithm to estimate three different photovoltaic model parameters of RTC France and PVM 752 GaAs thin film cells. A stochastic radial motion optimization (RMO) algorithm was employed by Ganesh et al. [19] to design the parameters of the single-diode and dual-diode models of solar photovoltaic cells. Beşkirli et al. [20] implemented a tree seed algorithm (TSA) and evaluated its performance on STM6-40/36 PV modules. Li et al. [21] compared the performance of various algorithms and their variants in three PV models and a PV module model. Yu et al. [22] designed a novel LNMHGS algorithm based on the basic hunger games search algorithm to simulate the parameter extraction of the PV model under different light irradiance and temperature. Madhiarasan et al. [23] applied the barnacle optimization algorithm (BMO) for the first time to identify the unknown parameters of three photovoltaic cell panels. Experimental results show that the proposed method can obtain high-precision and reliable results with a small number of iterations, greatly reducing the calculation time. A multi-strategy-learning-boosted colony predation algorithm (MLCPA) was achieved by Wang et al. [24] to address solar system parameter identification issues, which adopted two learning-based operators incorporated into a colony predation algorithm (CPA); experimental results show that the proposed method can reasonably estimate different PV models and show significant performance advantages. Yu et al. [25] proposed an improved grey wolf optimizer (SPGWO) with a superior subpopulation and inferior subpopulation that combines the Gaussian mutation and Levy flight strategies to accurately identify five different PV model parameters. A comprehensive analysis of the experimental results shows that the proposed method provides reliable and stable search results.

The Flower Pollination Algorithm (FPA), introduced by Xin-She Yang in 2012, draws its inspiration from the intricate dynamics of flower pollination observed in the natural world [26]. The FPA algorithm efficaciously blends the exploration and exploitation processes through probability switching to carry out diverse pollination behaviors, which has been widely used in various optimization problems because of its simple structure, fewer parameters and high execution efficiency. Bayesian fusion technology based on the flower pollination algorithm (FPA) and grey wolf optimizer (GWO) [27] are proposed by S et al. [28] to extract the maximum power of different configurations of real photovoltaic arrays. The simulation result proves that the method can reduce costs and improve the actual efficiency of PV panels through the analysis of different shading pattern. MBFPA based on butterfly optimization algorithm (BOA) and flower pollination algorithm is employed by Wang et al. [29] to solve five popular engineering problems. Neighborhood global learning-based flower pollination algorithm is introduced by Chen et al. [30] to deal with Unmanned Aerial Vehicle (UAV) Path Planning Problems.

While the FPA algorithm has demonstrated numerous dependable applications, its slow convergence speed and low accuracy in scheme evaluation for photovoltaic models are shortcomings that cannot be ignored. To this end, the present paper introduces the boosting flower pollination algorithm (BFPA) to enhance both the efficiency and reliability of the FPA when applied to the parameter extraction of photovoltaic models. In detail, the Gaussian distribution was developed to reduce execution time while ensuring the solution accuracy. In the exploitation process, a clustering strategy was introduced to use better-performing pollen to guide the search for worse-performing pollen to ensure the desired evolution of the group. Further chaotic elite-guided learning strategy was utilized to fully mine the favorable information around the current unfavorable pollen to improve the population quality. In addition, the adaptive boundary handling strategy is proposed to effectively alleviate the evolutionary difficulty caused by the local optimal boundary of multi-aggregated populations. In order to verify the effectiveness of the proposed strategy in solving photovoltaic model issues and to provide a fair and comprehensive evaluation of BFPA, BFPA is first compared with eight popular methods on RTC France photovoltaic cells and Photowat-PWP201 photovoltaic modules to verify the power control transfer levels of the proposed method. And then three commercial photovoltaic modules were then used under different extreme environmental conditions to verify the conversion efficiency of BFPA. The experimental findings demonstrate that the BFPA generated simulated data are strongly correlated with the corresponding actual data.

The main contributions of this paper are summarized as follows:

- A Boosting Flower Pollination Algorithm (BFPA) combining three novel strategies is designed to estimate unknown parameters of different photovoltaic cell/module models.
- The proposed method is used to identify unknown parameters of three PV cell/module models and the manufacturer’s PV module model.
- Compared to existing popular methods, comprehensive experiments on BFPA were conducted in a variety of different environments. The research results verified that BFPA can provide more excellent results, indicating the significant competitive advantage of the proposed method in PV systems.

The remainder of this paper is organized as below. Section 2 gives the Materials and Methods. Section 3 conducts the simulation experiments and gives a comprehensive analysis of the results. Section 4 focuses on the discussion of the results in Section 3. And the conclusions and future works are given Section 5.

## 2. Materials and Methods

### 2.1. Mathematical Models of Photovoltaic Systems

**Figure 1:**
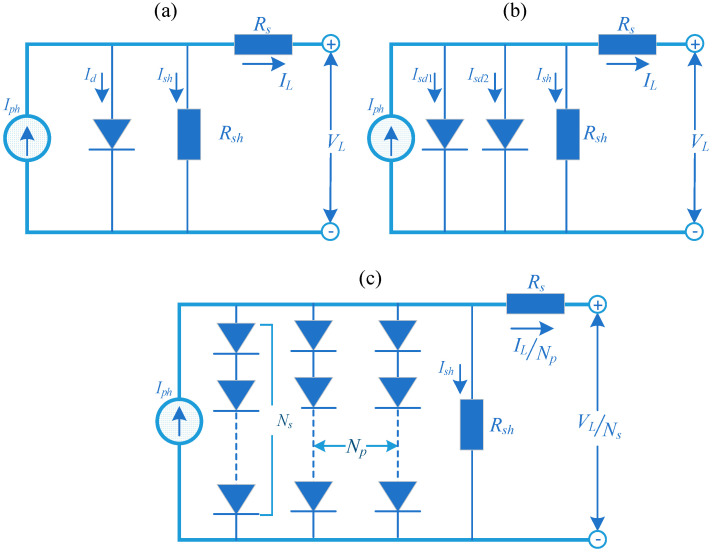
Sketch map of PMM.

Accurately identifying photovoltaic cell and module parameters is crucial for the operation of solar photovoltaic (PV) systems. To precisely determine the current and voltage characteristics of PV systems, commonly used mathematical models for single diodes (Figure 1a), double diodes (Figure 1b) and photovoltaic modules (Figure 1c) are adopted, as demonstrated below.

#### 2.1.1. Single Diode Model (SDM)

The single diode model (SDM) is currently the most popular and ideal model, which consists of a current source, a diode and two resistors, where the current source is connected in parallel with the diode, and the shunt resistor of the leakage current and the series resistor of the load current loss are further considered to ensure the complexity and stability of the model. According to the node current law of Kirchhoff, the output current of *I_L_* is defined as follows [31]:

(1) $$I_{L}=I_{ph}-I_{d}-I_{sh}$$

where *I_ph_* denotes the photocurrent, *I_d_* denotes the current through the diode, which can be obtained by Equation (2) according to the exponential curve equation of Shockley, and *I_sh_* denotes the current through the shunt resistor calculated by Equation (3).

(2) $$I_{d}=I_{sd}\cdot [\exp\left(\frac{q\cdot (V_{L}+R_{s}\cdot I_{L})}{n\cdot k\cdot T}\right)-1]$$

(3) $$I_{sh}=\frac{V_{L}+R_{s}\cdot I_{L}}{R_{sh}}$$

where *I_sd_* represents the diode reverse saturation current, *q* = 1.60217646 × 10^−19^ C represents the elementary charge, *V_L_* represents the output voltage, *R_s_* represents the series resistor, *n* represents the ideality factor of diode, *k* = 1.3806503 × 10^−23^ J/K represents the Boltzmann constant, *T* represents the Kelvin temperature. *R_sh_* represents the shunt resistor. Then combining Equations (1)–(3) to obtain the Equation (4):

(4) $$I_{L}=I_{ph}-I_{sd}\cdot [\exp\left(\frac{q\cdot (V_{L}+R_{s}\cdot I_{L})}{n\cdot k\cdot T}\right)-1]-\frac{V_{L}+R_{s}\cdot I_{L}}{R_{sh}}$$

It can be seen from Equation (4) that there are five unknown parameters (*I_ph_*, *I_sd_*, *R_s_*, *R_sh_*, *n*) that need to be identified to improve the stability of the photovoltaic system.

#### 2.1.2. Double Diode Model (DDM)

The SDM model assumes that the output voltage is constant within the range of variation and does not consider the influence of the recombination current loss in the depletion region [32]. In order to make the model more in line with actual needs, two diodes in parallel with a current source to simulate complex and rectified currents to obtain the double diode model (DDM). the output current *I_L_* of DDM is defined as follows [33]:

(5) $$\begin{array}{ll} I_{L} & =I_{ph}-I_{sd1}-I_{sd2}-I_{sh}\\ & =I_{ph}-I_{sd1}\left[\exp\left(\frac{q\cdot (V_{L}+R_{s}\cdot I_{L}}{n_{1}\cdot k\cdot T}\right)-1\right]-I_{sd2}\left[\exp\left(\frac{q\cdot (V_{L}+R_{s}\cdot I_{L}}{n_{2}\cdot k\cdot T}\right)-1\right]\\ & -\frac{V_{L}+R_{s}\cdot I_{L}}{R_{sh}} \end{array}$$

where *I_sd_*_1_ and *I_sd_*_2_ denote the diffusion and saturation currents of the diode, respectively. *n*_1_ and *n*_2_ denote the diffusion and saturation diode ideality factors, respectively. It can be seen from Equation (5) that there are seven unknown parameters (*I_ph_*, *I_sd_*_1_, *R_s_*, *R_sh_*, *n*_1_, *I_sd_*_2_, *n*_2_) that need to be estimated.

#### 2.1.3. PV Module Model (PMM)

The photovoltaic module model (PMM) is composed of several solar cells connected in series and/or in parallel, and the output current of PMM module can be defined as follows [34]:

(6) $$I_{L}=I_{ph}N_{p}-I_{sd}N_{p}\cdot \left[\exp\left(\frac{q\cdot (V_{L}/N_{s}+R_{s}\cdot I_{L}/N_{p})}{n\cdot k\cdot T}\right)-1\right]-\frac{V_{L}/N_{s}+R_{s}\cdot I_{L}/N_{p}}{R_{sh}N_{s}/N_{p}}$$

where *N_p_* and *N_s_* are the number of solar cells in parallel and series, respectively. Therefore, there are five unknown parameters that should be estimated, including *I_ph_*, *I_sd_*, *R_s_*, *R_sh_* and *n*.

### 2.2. Problem Formulation

In order to obtain accurate and reliable photovoltaic model parameters, the optimization objective is described by minimizing the difference between the experimentally observed current and the simulated current. The error value of the *k*th point of the observed current value and the simulated current value for different models can be expressed as follows [31,33,34]:

(7) $$\left\{\begin{array}{l} f_{k}(V_{L},I_{L},X)=I_{ph}-I_{sd}\cdot \left[\exp\left(\frac{q\cdot (V_{L}+R_{s}\cdot I_{L})}{n\cdot k\cdot T}\right)-1\right]-\frac{V_{L}+R_{s}\cdot I_{L}}{R_{sh}}-I_{L}\\ X=\{I_{ph},I_{sd},R_{s},R_{sh},n\} \end{array}\right.$$

(8) $$\left\{\begin{array}{l} \begin{array}{l} f_{k}(V_{L},I_{L},X)=I_{ph}-I_{sd1}\cdot \left[\exp\left(\frac{q\cdot (V_{L}+R_{s}\cdot I_{L})}{n_{1}\cdot k\cdot T}\right)-1\right]\\ \;\;\;\;-I_{sd2}\cdot \left[\exp\left(\frac{q\cdot (V_{L}+R_{s}\cdot I_{L})}{n_{2}\cdot k\cdot T}\right)-1\right]-\frac{V_{L}+R_{s}\cdot I_{L}}{R_{sh}}-I_{L}\text{​} \end{array}\\ X=\{I_{ph},I_{sd1},R_{s},R_{sh},n_{1},I_{sd2},n_{2}\} \end{array}\right.$$

(9) $$\left\{\begin{array}{l} \begin{array}{l} f_{k}(V_{L},I_{L},X)=I_{ph}N_{p}-I_{sd}N_{p}\cdot \left[\exp\left(\frac{q\cdot (V_{L}/N_{s}+R_{s}\cdot I_{L}/N_{p})}{n\cdot k\cdot T}\right)-1\right]\\ \;\;\;\;\;\;-\frac{V_{L}/N_{s}+R_{s}\cdot I_{L}/N_{p}}{R_{sh}\cdot N_{s}/N_{p}}-I_{L}\text{​} \end{array}\\ X=\{I_{ph},I_{sd},R_{s},R_{sh},n\} \end{array}\right.$$

Then the overall error at the observed and simulated current can then be quantified by the root mean square error (RMSE) as the objective function [35]:

(10) $$\mathrm{RMSE}(X)=\sqrt{\frac{1}{K}\overset{K}{\underset{k=1}{\sum }}f_{k}{\left(V_{L},I_{L},X\right)}^{2}}$$

where ***X*** denotes the unknown variable of the problem, *K* denotes the number of the observed current-voltage data.

### 2.3. Boosting Flower Pollination Algorithm (BFPA)

#### 2.3.1. Summary of FPA

**Figure 2:**
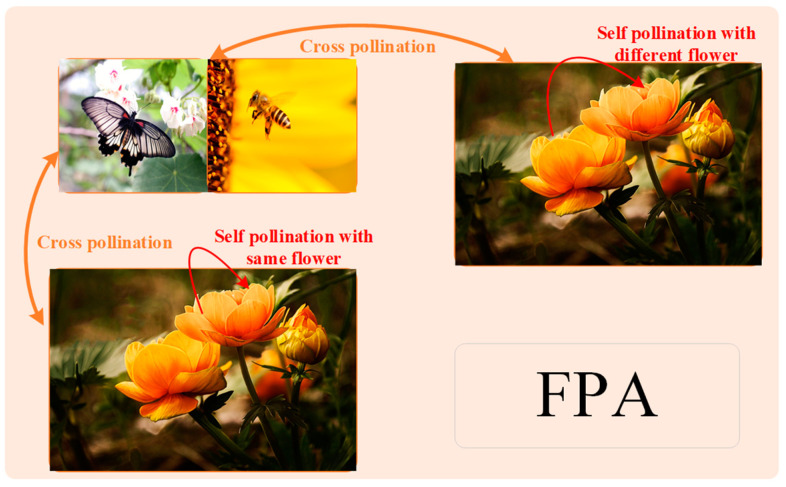
The schematic diagram of FPA.

The flower pollination algorithm (FPA), as shown in Figure 2, is a biomass-inspired algorithm based on the pollination behavior of the flowers [26]. Pollination behavior mainly includes two ways: self-pollination and cross-pollination. Self-pollination means using pollen from the same flower or from different flowers of the same plant when there is no reliable pollinator, while cross-pollination is when pollinators such as insects or birds carry pollen for long distances flights to cause pollination between different plans. In FPA, the exploration and exploitation behavior of the algorithm is guaranteed through four idealized principles to ensure that the algorithm can search for a better solution. The principles are as follows:

(1) Biological pollination and cross-pollination carry out the global pollination processes through the Lévy flight behavior.
(2) Abiotic and self-pollination perform local pollination process via their own characteristics.
(3) Flower constancy, also known as reproductive rate, can be considered to be related to the similarity between two flowers.
(4) Local pollination and global pollination can be switched freely and controlled by probability *p* ∈ [0, 1].

Combining the above rules, the mathematical model of the FPA algorithm is defined as follows:

To ensure that FPA has an efficient search ability, the random initialization strategy is selected to generate the initial population:

(11) $$\left\{\begin{array}{l} X_{ij}^{t}=(Ub_{j}-Lb_{j})\cdot r_{1}+Lb_{j}\\ i\in [1,N],j\in [1,D] \end{array}\right.$$

where $X_{ij}^{t}$ is the position of *j*th dimension for *i*th pollen at *t*th iteration, *Ub_j_* and *Lb_j_* are the upper and lower boundaries of *j*th dimension of the problem, respectively. *r*_1_ is the random number generated within [0, 1]. *N* is the number of pollens in the population, *D* is the number of variables in each pollen.

For the global pollination of rule (1) combined with flower constancy of rule (3) can be defined mathematically as:

(12) $$X_{ij}^{t+1}=X_{ij}^{t}+(X_{ij}^{t}-G_{j}^{t})\cdot Levy\left(s,\lambda \right)$$

where $G_{j}^{t}$ represents the global best position of *j*th dimension in the pollen population. *Levy*(*s*, *λ*) represents the search step size corresponding to Lévy flight, which is defined as follows:

(13) $$Levy\left(s,\lambda \right)\sim \frac{\lambda \cdot \Gamma \left(\lambda \right)\sin\left(0.5\cdot \pi \lambda \right)}{\pi }\cdot \frac{1}{s^{1+\lambda }},\;\left|s\right|\to \infty$$

where Γ(*λ*) represents gamma function with parameter *λ*. *s* represents the random step size generated by nonlinear transformation, as follows:

(14) $$\left\{\begin{array}{l} s=\frac{u}{{\left|v\right|}^{\lambda^{-1}}}\\ u∼Gauss\left(0,\sigma_{}^{2}\right)\;,v∼Gauss\left(0,1.0\right) \end{array}\right.$$

where *u* and *v* are the random number generated by a normal distribution with mean 0 and standard deviation σ and 1.0 respectively. And the σ can be expressed as follows:

(15) $$\sigma_{}={\left[\frac{\Gamma \left(1+\lambda \right)}{\lambda \cdot \Gamma \left[\left(1+\lambda \right)\cdot 0.5\right]}\times \frac{\sin\left(0.5\cdot \pi \lambda \right)}{2^{(\lambda -1)\cdot 0.5}}\right]}^{1/\lambda }$$

where sin(·) represents the sine function.

For the local pollination of rule (2) combined with flower constancy of rule (3), the formula mathematically expressed as follows:

(16) $$X_{ij}^{t+1}=X_{ij}^{t}+(X_{l_{1}j}^{t}-X_{l_{2}j}^{t})\cdot r_{2}$$

where $X_{l_{1}j}^{t}$ and $X_{l_{2}j}^{t}$ are the position of *j*th dimension for *l*1th and *l*2th pollens at *t*th iteration, *l*_1_ and *l*_2_ are the random index of pollens. *r*_2_ is the number randomly generated within [0, 1].

For the rule (4), local and global pollination can occur in all pollen populations, where not-so-far-away flower have a greater probability of local pollination, while adjacent and farther flower blocks have a smaller probability of global pollination, as follows:

(17) $$\left\{\begin{array}{l} \mathrm{Local}\;\mathrm{pollination},\;r_{3}>p\\ \mathrm{Global}\;\mathrm{pollination},\;r_{3}\leq p \end{array}\right.$$

where $r_{3}$ represents the random number in [0, 1], *p* controls the probability of switching between local pollination and global pollination, and 0.8 is recommended in the original literature [36].

#### 2.3.2. The Proposed Algorithm of BFPA

Metaheuristic algorithms try to make the population find an appropriate balance between exploration and exploitation in the process of optimization. The exploration process is to allow individuals to have more opportunities to search for location areas in the problem space on a large scale, while the exploitation process is to use the information around individuals as much as possible to find more favorable locations.

In the original FPA, the global pollination adopts Lévy flight behavior, which can ensure the pollinators can fly and move in a longer range, but the excessive flight step size will reduce the robustness and increase the CPU execution time of FPA. Local pollination does not consider the performance difference between pollinators in the process of selecting individuals, which may lead to the generation of individuals that are not conducive to evolution. Based on the above analysis, in this paper, the boosting flower pollination algorithm is proposed to improve the performance of original FPA in parameter identification of photovoltaic systems. In detail, the Gaussian distribution is adopted to replace the Lévy flight distribution, which saves the increased computing resources due to the calculation of the Lévy distribution process while ensuring that the population has a better global pollination ability. Moreover, the population is firstly divided according to the fitness of the individual, and then the individual with poor performance learns from the individual with better performance in random individual selection, which ensures the diversity of the population and improves the convergence speed. In addition, during the whole search process, poor individuals learn with the help of favorable position information in the population, which effectively improves the performance of the population. For the pollen in the population that exceeds the boundary of the problem, the adaptive boundary handling strategy is used to correct the pollen behavior. The main idea of BFPA is expressed as follows.

##### Gaussian Distribution Global Pollination Strategy

In the global pollination process, the pollen population uses Lévy random walk for long-distance flight. Although it can ensure that the population jumps out of the local optimal position in time, it may cause the algorithm to fail to find a more stable solution during repeated experiments. For this reason, the Gaussian distribution [37] is adopted to replace the complex Lévy flight distribution, which can effectively avoid optimization stagnation. At the same time, the tail of the Gaussian distribution is smooth, which ensures the stability of the algorithm in the search process, as follows:

(18) $$X_{ij}^{t+1}=X_{ij}^{t}+Gauss\left(0,\alpha \right)\cdot (G_{j}^{t}-X_{ij}^{t})$$

where *Gauss*(0, *α*) represents the Gaussian distribution random number with mean 0 and standard deviation *α*.

##### The Clustering Strategy of Population

**Figure 3:**
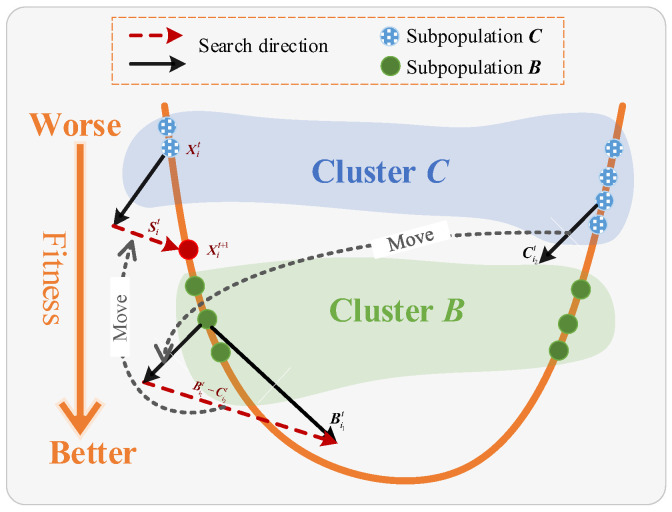
The clustering strategy of population.

In the population-based algorithm, individuals with poor performance search for individuals with good performance to have more chances to find favorable areas, but FPA does not consider pollen performance in the random selection of local pollination. For this reason, a strategy based on population clustering [38] is proposed to ensure that the population evolves in a favorable direction. To be specific, calculate the fitness of the pollen population and sort it from good to bad, then record the first *N*/2 pollen with better performance as subpopulation ***B***, and the remaining pollen with poor performance as subpopulation ***C***, that is to say, the fitness of any one in ***B*** is better than the pollen in the ***C***, the detail process is given by Algorithm 1, as shown in Figure 3, the mathematical expression is defined as follows:

(19) $$\left\{\begin{array}{l} X_{i}^{t+1}=X_{i}^{t}+S_{i}^{t}\\ S_{i}^{t}=r_{4}\cdot (B_{i_{1}}^{t}-C_{i_{2}}^{t}) \end{array}\right.$$

where $S_{i}^{t}$ denotes the favourable search direction vector of *i*th pollen at *t*th iteration. $B_{i_{1}}^{t}$ denotes the position vector of the *i*1th pollen randomly selected from subpopulation ***B*** at *t*th iteration, $C_{i_{2}}^{t}$ denotes the position vector of the *i*2th pollen randomly selected from subpopulation ***C*** at *t*th iteration, *r*_4_ denotes the random number within [0, 1].
**Algorithm 1:** Pseudo code of clustering strategy of population.**If** *r*_3_ ≥ *p* then  Cluster pollen populations according to fitness to get better subpopulation ***B*** and poor subpopulation ***C***  Randomly select an individual from ***B*** and ***C*** respectively and denote the indices *i*_1_ and *i*_2_.  Perform Equation (19) to obtain $X_{i}^{t+1}$  Calculate the fitness of $X_{i}^{t+1}$ and $X_{i}^{t}$  **If** $F(X_{i}^{t+1})\leq F(X_{i}^{t})$ **then**//F(⋅) denotes the fitness function   Let $X_{i}^{t}$ = $X_{i}^{t+1}$  **End if****End if**

##### The Chaotic Elite-Guided Learning Strategy

In the process of population evolution, the global best position of the population and the personal best position of the pollen are two important parameters. The global best position saves the optimal solution currently found by the population, while the personal best position records the favourable position found by each pollen evolution to the current stage. In order to make the population have better development performance in the evolution process, a random and ergodic chaotic sequence is introduced on the basis of the global best position and the personal best position to realize the refined search near the favourable position area, given in Algorithm 2. In the early stage, it is necessary to make full use of the information around itself for large-scale exploration, and in the later stage of the search, it is necessary to obtain more information near the best pollen. Then, if the new pollen produced performs better than the original pollen, the new individual is replaced. It should be noted that in order to effectively improve the pollen with poor performance in the population, the strategy proposed in this section is implemented in the subpopulation ***B*** proposed in the previous section.

(20) $$z_{l+1}=z_{l}\cdot \eta \cdot (1-z_{l})$$

(21) Xi3jt=Gjt+step⋅2⋅zl−1, if(r5<FesFesmax)Pi3jt, else

where $z_{l}$ denotes the chaos value at *l*th iteration, *r*_5_ denotes the control degree of chaos, η denotes the random number, η∈[3.57,4.0] is recommended in [39]. *step* and *r*_5_ denote the random number in [0, 1], *Fes* and *Fes*_max_ denote the number of current functional evaluation and total functional evaluations, respectively. $P_{i_{3}j}^{t}\;$ denotes the personal best position of *j*th dimension of *i*3th pollen at *t*th iteration, *i*_3_ ∈ *B*.
**Algorithm 2:** Pseudo code of chaotic elite-guided learning strategyInitialize the random number η, and get z_0_ within [0, 1]Randomly select a pollen from subpopulation ***B*** and record the index as *i*_3_**For** *j* = 1 to *D* **do**  Calculate z*_l_*_+1_ by Equation (20)  **If** *r*_5_ < *Fes/Fes*_max_ **then**     Let $X_{i_{3}j}^{t}=G_{j}^{t}+step\cdot \left(2\cdot z_{l}-1\right)$  **Else**     $X_{i_{3}j}^{t}=P_{i_{3}j}^{t}\;$  **End if****End for**

##### Adaptive Boundary Handling Strategy

One of the means to ensure that the algorithms have efficient search performance are to properly and effectively handle boundary constraints [40]. Multimodal issues usually include multiple local optimal value near the boundaries of the problem, which will cause FPA to gather multiple pollen gathering in the process of searching for the optimal solution to make the population suspension at the local optimal position. To this end, the adaptive boundary handling strategy is proposed, given in Algorithm 3. Specifically, when the updated individual location exceeds the search range, if the excess value is within the pre-set threshold, it is processed according to the mirroring boundary processing method [41], otherwise the individual returns to the closest problem boundary, as follows:

(22) $$X_{ij}^{t}=\left\{\begin{array}{l} 2\cdot Lb_{j}-X_{ij}^{t}\;,\left(Lb_{j}-X_{ij}^{t}\right)\leq \phi \\ Lb_{j}\;\;\;\;\;,else \end{array},X_{ij}^{t}<Lb_{j}\;\right.$$

(23) $$X_{ij}^{t}=\left\{\begin{array}{l} 2\cdot Ub_{j}-X_{ij}^{t}\;,\left(X_{ij}^{t}-Ub_{j}\right)\leq \phi \\ Ub_{j}\;\;\;\;\;,else \end{array},X_{ij}^{t}>Ub_{j}\right.$$

where ϕ denotes the pre-set threshold, and ϕ = (*Ub_j_* + *Lb_j_*) × 0.5.
**Algorithm 3:** Pseudo code of adaptive boundary handling strategy**For** *j* = 1 to *D* **do**  Let *ϕ* = (*Ub_j_* + *Lb_j_*) × 0.5  **If** *X_ij_* > *Ub_j_* **then**     **If** (*X_ij_* − *Ub_j_*) < *ϕ*       *X_ij_* = 2 × *Ub_j_* − *X_ij_*     **Else**       *X_ij_* = *Ub_j_*     **End if**   **Else if** *X_ij_* < *Lb_j_* **then**     **If** (*Lb_j_* − *X_ij_*) < *ϕ*       *X_ij_* = 2 × *Lb_j_* − *X_ij_*     **Else**       *X_ij_* = *Lb_j_*     **End if**  **End if****End for**

##### The Framework of BFPA

**Figure 4:**
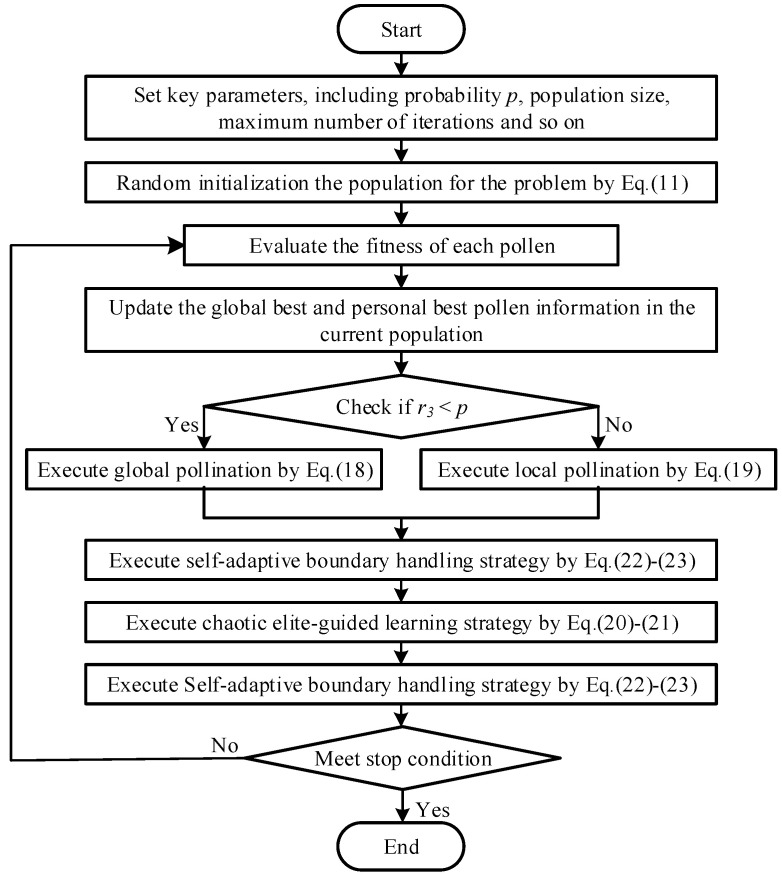
The execution flowchart of BFPA algorithm.

Based on the above detailed description, Figure 4 gives the execution flowchart of BFPA. It can be seen that compared with the original FPA, the complexity of BFPA does not increase significantly.

## 3. Simulation Results

### 3.1. Results of PV Cell and Module Model

In this section, the single-diode model (SDM), double-diode model (DDM) and photovoltaic module model (PMM) are used to verify the performance of the algorithm in the identification of photovoltaic parameters. The experiment selects the well-known current-voltage(*I*-*V*) data in the literature [42] as benchmark. On the one hand, 26 pairs of current and voltage data observed by the commercial RTC France photovoltaic cell with a diameter of 57 mm at an irradiation intensity of 1000 W/m^2^ irradiance and 33 °C temperature is used as the SDM and DDM data sets. On the other hand, 25 pairs of current and voltage values observed with the Photowat-PWP201 module under the irradiance of 1000 W/m^2^ at 45 °C is adopted as PMM data set. In order to ensure the fairness of the experiment, the upper and lower limits of the unknown parameters that need to be identified are kept consistent with the original literature, as shown in Table S1.

BFPA is compared with a variety of methods according to the single variable principle to eliminate the adverse effects of the environment and parameter settings on the experiment. The methods that are more popular and have superior performance in photovoltaic parameter identification problems are introduced into the experiment, including cooperation search algorithm (CSA) [43], marine predators algorithm (MPA) [44], RIME [45], backtracking search optimization algorithm (BSA) [46], generalized oppositional teaching learning based optimization (GOTLBO) [47], adaptive differential evolution with optional external archive (JADE) [48], performance-guided JAYA algorithm (PGJAYA) [49]. And flower pollination algorithm (FPA) [36] is also used to illustrate the effectiveness of the proposed strategy. Table S2 gives the detailed parameter settings of the adopted algorithm. Furthermore, in order to ensure the fairness of the experiment and facilitate the statistical comparison, the maximum function evaluations and the number of independent experiments of each method for different photovoltaic parameter identification problems are set to 30,000 and 30, respectively.

Further, simulation experiments were carried out using Multi-crystalline (KC200GT), Mono-crystalline (SM55) and thin-film (ST40) photovoltaic modules under different temperatures and various light irradiances. All the experiments were simulated in personal computer with 32 GB RAM and Intel(R) Core (TM) CPU i7-11800H @ 2.30 GHz under Windows10 (64 bit), and all the tests were performed on JAVA programming language.

In the experiment, the absolute error and relative error of current can be calculated through the observed and simulated *I*-*V* values, as defined follows:

(24) $$\left\{\begin{array}{l} IA_{e}=I_{o}-I_{s}\\ IR_{e}=\frac{I_{o}-I_{s}}{I_{o}}\cdot 100\% \end{array}\right.$$

where *IA_e_* and *IR_e_* denote the absolute error value and relative error value of current, respectively; $I_{o}$ and $I_{s}$ are the observed current value and simulated current value, respectively.

Likewise, the absolute errors of power can be expressed as follows:

(25) $$WA_{e}=W_{o}-W_{s}$$

where $WA_{e}$ denotes the absolute error value of power; $W_{o}$ and $W_{s}$ are the observed power value and simulated power value, respectively.

#### 3.1.1. Statistical Results Analysis on Different PV Models

In this subsection, BFPA is compared with the control method to verify the stability, reliability and convergence efficiency of the proposed method. Table S3 shows the statistical results of different methods after running 30 times, including the best, median, mean, worst and STD (standard deviation). The best results of different indicators are displayed in bold.

It can be seen from Table S3 that in the SDM, DDM and PMM problems, the proposed method can obtain more stable and higher precision results than the control method in all statistical indicators. Specifically, for SDM, JADE and PGJAYA are similar to the results of the proposed method in the best indicators. But considering statistical indicators such as median, mean and worst, its performance is worse than BFPA results. Especially in the comparison of STD indicators, BFPA is 13 orders of magnitude more stable than other methods. For DDM, although the statistical results of BFPA and other methods are similar, the result index of BFPA is also the best. For the PV parameter identification problem, any improvement in accuracy can be considered as a more reliable solution. In the case of PMM, PGJAYA can get the second-best results in the best, median, while JADE can get the second-best results in the mean, worst and STD.

Table S4 gives the results of the Wilcoxon signed-rank test [50] under the 5% confidence interval of significance. It can be seen that the proposed method can defeat other control methods in the comparison of the three model results. At the same time, it can be seen from the last column that the superiority of BFPA is trustworthy.

**Figure 5:**
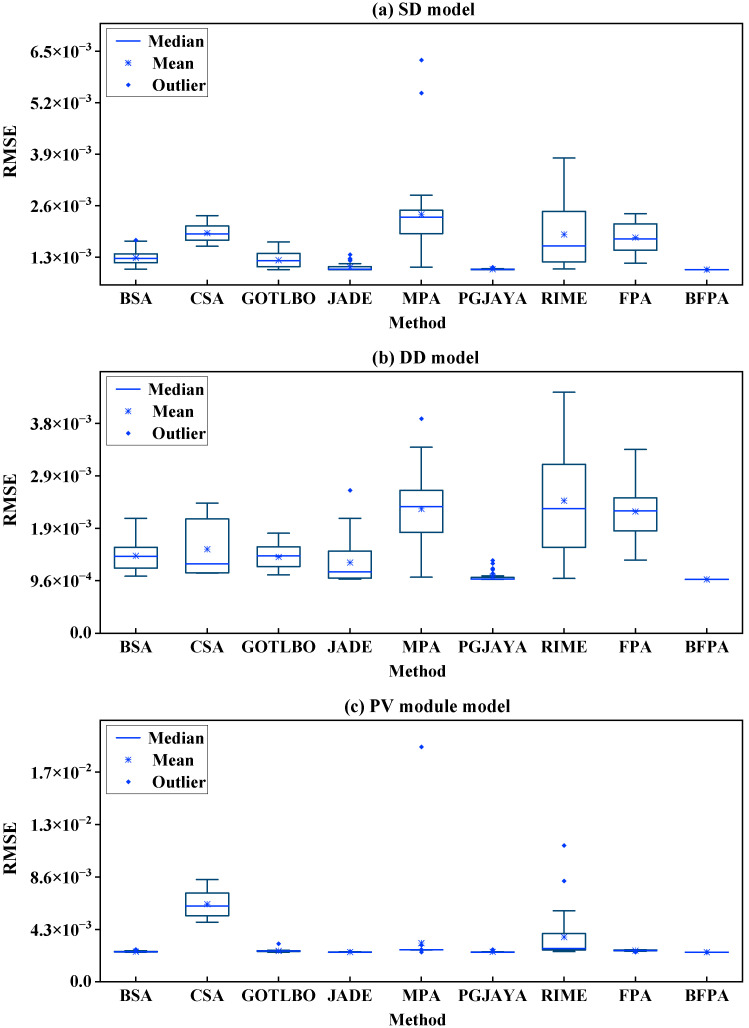
The boxplot of different methods for SDM, DDM and PMM.

In addition, in order to more intuitively show the dispersion of the RMSE of different methods running 30 times, a box plot is drawn in Figure 5. It can be seen that for SDM and DDM, except for PGJAYA and BFPA, the data distribution of other control methods presents different levels of distribution fluctuations, and MPA, JADE and PGJAYA also appear different degrees of outliers, while the data distribution of the proposed method is dense and stable. For PMM, the data distribution outside CSA and RIME is relatively poor, and the overall distribution of other methods is relatively stable. From the overall distribution of the box plot, the proposed BFPA method has excellent reliability.

**Figure 6:**
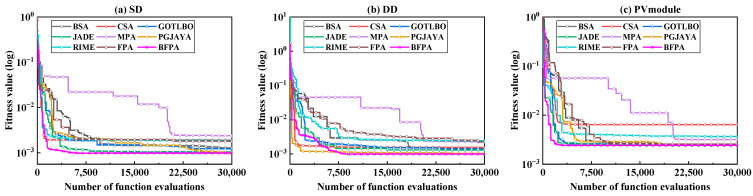
The number of function evaluation of multiple methods.

The average convergence curve of different methods running independently multiple times is further shown in Figure 6. It can be seen that for SDM and PMM, the proposed BFPA method has a faster convergence speed; For DDM, BFPA converges slowly in the early stage and gradually finds a better advantage area than the control method as the search progresses.

**Figure 7:**
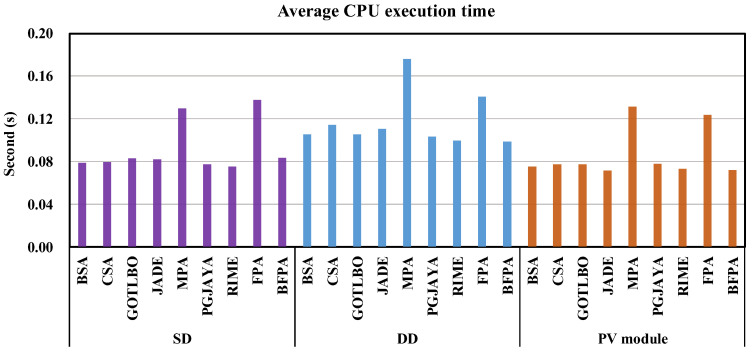
The average CPU execution time of different methods.

Moreover, Figure 7 shows the average CPU execution time of all methods under different problems. On the whole, the execution time of MPA and FPA is relatively long, and the execution time of BFPA and other control methods is similar because BFPA does not need to carry out Lévy flight calculations like the original FPA. The above analysis implies that BFPA can obtain satisfactory computational solutions in a time similar to state-of-the-art algorithms.

#### 3.1.2. Result on Single Diode Model

This section discusses the experimental results of optimal parameters of different methods under the SDM problem. Table S5 shows the five parameters to be estimated and the RMSE results. As can be seen from Table S5, BFPA obtained the best model parameter optimization results, followed by PGJAYA and JADE methods. Moreover, the model parameter results obtained by CSA, MPA and FPA are quite different from other methods, which shows that it is difficult for these three types of methods to obtain more reasonable model parameters for the SDM problem. From this, it can be concluded that the BFPA method can effectively identify unknown model parameters in SDM.

**Figure 8:**
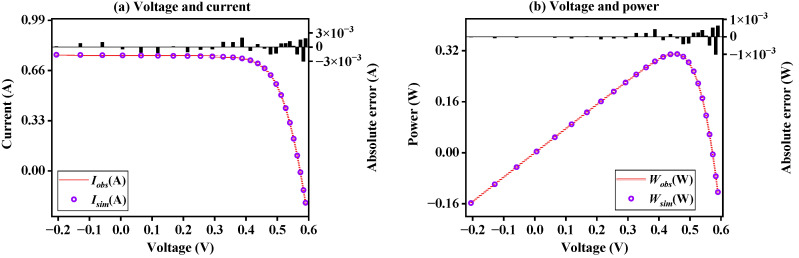
The *I*-*V* and *P*-*V* characteristics curve on SDM.

Figure 8 shows the *I*-*V* and *P*-*V* curves generated after the model parameters are obtained through BFPA under SDM. It can be seen that the simulation data obtained through the model parameters can be highly fitted to the observed data. Results shows that the proposed BFPA method can accurately identify SDM problems.

Table S6 further shows the statistical results of current and power errors of BFPA on SDM. It can be seen that the absolute error value of the current is between −2.50741270 × 10^−3^ and 1.61722183 × 10^−3^, and the absolute error value of the power is between −1.46257383 × 10^−3^ and 9.01327437 × 10^−4^. The results again illustrate the accuracy of BFPA parameter identification.

#### 3.1.3. Result on Double Diode Model

Table S7 shows the statistical results and RMSE values of the optimal values of the seven recognition parameters obtained by various methods under the DDM problem. For the DDM case, BFPA, JADE and PYJAYA perform better than other methods, among which BFPA is more prominent because it has a better RMSE value. The analysis of the above results shows that the proposed BFPA has greater competitiveness than other methods.

**Figure 9:**
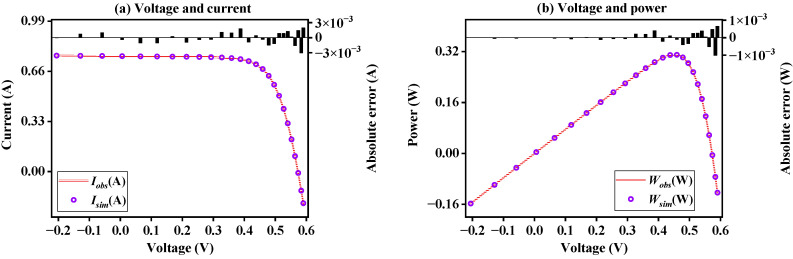
The *I*-*V* and *P*-*V* characteristics curve on DDM.

Figure 9 shows the fitting degree of *I*-*V* and *P*-*V* curves of BFPA on the DDM case. It can be seen that the data results obtained by simulation are highly matched with the provided observed data results. The above shows that the BFPA method can also better estimate the parameters of the DDM problem.

In order to illustrate the accuracy of the BFPA method, the error results of the observed data and the simulated data are further shown in Table S8. It can be seen from the table that the current absolute error value is between −2.54343465 × 10^−3^ and 1.62841193 × 10^−3^, and the power absolute error value between −1.48358543 × 10^−3^ and 9.60763041 × 10^−4^. Compared with the optimal parameters obtained by SDM, the error is relatively large because DDM needs to identify more parameters. However, the current error range can still reflect the accuracy of BFPA for extracting DDM parameters.

#### 3.1.4. Result on Photovoltaic Module Model

**Figure 10:**
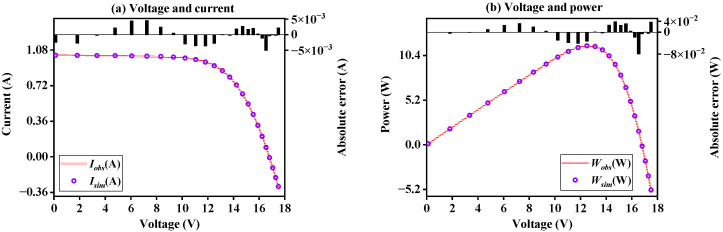
The *I*-*V* and *P*-*V* characteristics curve on PMM.

This subsection adopts BFPA and other control methods to obtain five unknown parameters and RMSE values on the *PV* module model, as shown in Table S9. It can be seen that BFPA has obtained a more ideal RMSE value than other control methods. Figure 10 draws the characteristic curves of *I*-*V* and *P*-*V* generated by the optimal parameters of BFPA. It can be seen that the obtained simulation data is highly consistent with the observed data, showing the superior performance of the algorithm in the PMM case. Table S10 further displays the absolute error value of current and power on the PMM case, it can be seen from the table that the error between the results obtained by BFPA simulation and the observed results is very small, which once again shows that BFPA can accurately identify unknown PMM parameters.

### 3.2. The Result of Datasheets from Different Manufacturers

In order to further verify the practicality and reliability of the proposed method in various environments, three widely accepted photovoltaic modules, KC200GT, SM55 and ST40 [51], were used to verify the performance of BFPA. The experimental data used is directly extracted from the *I*-*V* data under different irradiation and temperature in the datasheet from different manufacturers. It should be pointed out that extracting parameters from the *I*-*V* data given in the manufacturer’s datasheets, it will help us better understand the performance of different photovoltaic products.

Unlike the PV model described earlier, the model established in this subsection has variable boundary parameters. The current *I_ph_* is bounded by the short circuit current *I_sc_* and the temperature coefficient *α* [2]. The short-circuit current *I_sc_* under non-standard experimental conditions is shown as follows:

(26) $$I_{SC}\left(G,T\right)=I_{SC\_STC}\times \frac{G}{G_{STC}}+\alpha \cdot \left(T-T_{STC}\right)$$

where $I_{SC}\left(G,T\right)$ denotes the short circuit current at the *G* W/m^2^ irradiance light and *T* °C temperature. $I_{SC\_STC}$ and $T_{STC}$ stand the short circuit and temperature at standard test condition.

The upper and lower boundary of the model extraction unknown parameters are set as: *I_ph_* (A) = [0, 2*I_sc_*], *I_sd_* (μA) = [0, 100], *R_s_* (Ω) = [0, 2], *R_sh_* (Ω) = [0, 5000], *n* = [1, 4].

**Figure 11:**
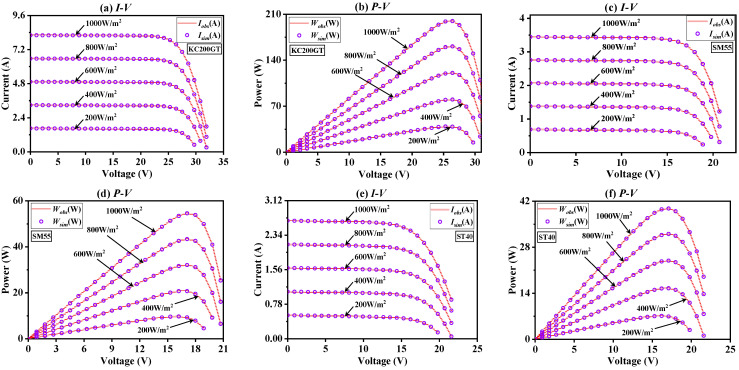
The *I*-*V* and *P*-*V* characteristics curve of three PV modules at diverse irradiance.

Table S11 shows the model parameter extraction results of three photovoltaic modules obtained by BFPA under multi-level light irradiance (200 W/m^2^, 400 W/m^2^, 600 W/m^2^, 800 W/m^2^ and 1000 W/m^2^) at 25 °C. It can be concluded that BFPA can always maintain a small RMSE value under different irradiances of the same model, which shows that the proposed method has good stability and reliability. Figure 11 further displays the characteristic curves of *I*-*V* and *P*-*V* after the model parameters are obtained. It can be clearly seen from the figure that for different irradiance, the proposed method can obtain almost the same simulated values as the observed data from the three models. In summary, BFPA can effectively solve the problem of parameter identification of PV module models under different irradiation levels and is an effective solution tool.

**Figure 12:**
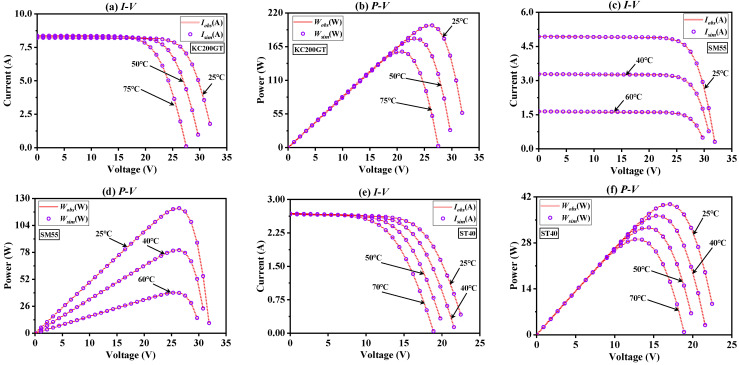
The *I*-*V* and *P*-*V* characteristics curve of three PV modules at different temperatures.

Furthermore, the irradiance was fixed at 1000 W/m^2^, and the experimental temperature was changed to simulate the recognition level of BFPA to three unknown parameters of photovoltaic modules. Table S12 shows the results of photovoltaic unknown parameter extraction obtained by BFPA at various temperatures. It can be seen that BFPA can obtain more ideal RMSE values at different temperatures. Further, the *I*-*V* and *P*-*V* fitting results of simulated data and observed data at multiple temperatures are plotted in Figure 12. It can be concluded that the simulated data and the observed data have a high level of fitting. The above shows that even under the temperature change scenario, BFPA can still accurately identify the parameters of photovoltaic modules, which is a reliable method.

## 4. Discussions

Three novel learning strategies are introduced into the standard FPA method to improve its own performance defects in photovoltaic cell/module parameter identification. The study uses SDM, DDM and PVM models and uses multiple sets of real current-voltage data sets to fully verify the performance of the proposed method. Experimental results show that the proposed BFPA can extract photovoltaic cell/module parameters with good accuracy from *I*-*V* data and is an excellent candidate.

According to Section 3.1, The proposed BFPA method is used to identify unknown parameters of three different PV models. First, in the analysis of statistical results, the proposed method can achieve the best performance in Best, Median, Mean, Worst and STD indicators, and shows ultra-high significance in Wilcoxon sign rank. According to the box plot test results, compared with other control methods, the BFPA method has stable results after repeated runs and greatly improved performance. At the same time, it can be seen from the fitted *I*-*V* curve and *P*-*V* curve that the optimization results of the proposed method are highly consistent with the actual measured results. This means that BFPA can be used to solve the problem at hand as a reference alternative to control methods. In addition, Section 3.2 is used to study the effectiveness of the proposed BFPA method in non-standard harsh environments. Datasheets from three photovoltaic manufacturers, KC200GT, SM55 and ST40, are selected, and a variety of experiments with different temperatures and light irradiances are conducted. The results show that the stability of BFPA results can be maintained in a satisfactory state. In summary, BFPA can be considered as an effective and stable potential method for identifying optical radiation model parameters.

In actual production, the proposed method can be used to analyze the parameter identification results of the *I*-*V* data of photovoltaic cells/modules, and the photovoltaic system control strategy can be adjusted based on the results to maximize energy output. Second, model parameters can be used to guide the selection of the type and size of photovoltaic panels most suitable for a specific application, which helps ensure that the PV system performs optimally under different conditions.

In addition, the limitations of the research in this article are also worth noting. First, although the proposed method saves computational time compared to the original method, the proposed strategy increases the number of hyperparameter settings. Secondly, the established model considers temperature and light irradiance changes over time in a fixed situation, which is not enough for actual dynamic environment applications. Finally, the open-source dataset used in the study is limited, and more data sets need to be obtained from actually used photovoltaic products to verify the effectiveness of the method.

## 5. Conclusions and Future Works

Accurately extracting unknown parameters of photovoltaic cells and module models is of great significance in the field of power systems. In this paper, the boosting flower pollination algorithm (BFPA) is proposed to estimate the parameters of various photovoltaic models. In BFPA, the gaussian global pollination strategy ensures the search stability and prevents premature convergence of the population. The clustering strategy of the population uses the fitness of the current population as the criterion for clustering search, which can effectively explore favorable positions in the problem space. In addition, in order to ensure that more favorable search directions can be provided for the population, a chaotic elite-guided learning strategy was introduced to search around the pollen with poor performance. Finally, the adaptive boundary handling strategy effectively alleviates the negative impact of population search due to the invalid boundary aggregation of the population. In order to evaluate BFPA effectively, a large number of simulation experiments are carried out using various photovoltaic models composed of single diode, double diode and photovoltaic module models. The proposed method has been thoroughly evaluated in comparison to several well-established methods in the field. The experimental results unequivocally demonstrate that our approach is not only reliable but also highly effective and robust. Moreover, it exhibits a noteworthy level of competitiveness when compared to its counterparts. Finally, the proposed method was tested on ST40, KC200GT and SM55 photovoltaic modules under different lighting conditions and temperatures. The results show that the extraction results of BFPA can perfectly fit the actual measurement data. On a global scale, the recommended methods can help researchers analyze the performance of photovoltaic models more quickly, enhance the reliability of photovoltaic systems through real-time monitoring and parameter adjustments, reduce failures and downtime, and contribute to the broader promotion and application of solar energy technology worldwide.

In future studies, we extend the proposed method to extend to the dynamic PV problem. In addition, we will try to apply BFPA to renewable energy problems with complex constraints and multiple objectives.

## Data Availability

Not applicable.

## Supplementary Materials

The following supporting information can be downloaded at: https://www.mdpi.com/article/10.3390/s23198324/s1. Table S1: The ranges of dentification parameters on different models; Table S2: Parameters settings of the selected methods; Table S3: The statistical results of different methods; Table S4: The Wilcoxon signed rank test with α = 5%; Table S5: The optimal parameter results for SDM; Table S6: The error value of current and power on SDM; Table 7: The optimal parameter results for DDM; Table S8: The error value of current and power on DDM; Table S9: The optimal parameter results for PMM; Table S10: The error value of current and power on PMM; Table S11: The optimal parameter optimized by BFPA at diverse irradiance under 25 °C; Table S12: The optimal parameter optimized by BFPA at different temperatures under 1000 W/m^2^.

## Nomenclature

BFPA	Boosting flower pollination algorithm	*R_sh_*	Shunt resistor
FPA	Flower Pollination Algorithm	*N_p_*/*N*_*s*_	Number of solar cells in parallel/series
PV	Photovoltaic	***X***	Unknown variable of the problem
*I*-*V*	Current-voltage	*K*	Number of the observed current-voltage data
SDM	Single diode model	RMSE	Root mean square error
DDM	Double diode model	Γ(*λ*)	Gamma function
PMM	Photovoltaic module model	sin(·)	Sine function
*I_ph_*	Photocurrent	*Gauss*(0, *α*)	Gaussian distribution function
*I_d_*	Current through the diode	*Levy*(*s*, *λ*)	Step size of Lévy flight
*I_sh_*	Current through the shunt resistor	*N*	Number of pollens
*I*_sd_, *I_sd_*_1_*, I_sd_*_2_	Currents of diode	*D*	Number of variables
*q*	Elementary charge	***B***, ***C***	Subpopulation
*V_L_*	Output voltage	KC200GT	Multi-crystalline PV module
*R_s_*	Series resistor	SM55	Mono-crystalline PV module
*n, n*_1_ *, n*_2_	Diode ideality factors	ST40	Thin-film PV module
*k*	Boltzmann constant	*IA_e_*	Absolute error
*T*	Kelvin temperature	*IR_e_*	Relative error
