# Supplementary material for: A Parameter Estimation of Photovoltaic Models Using a Boosting Flower Pollination Algorithm
Source: Sensors (Basel). 2023 Oct 8;23(19):8324. doi: 10.3390/s23198324 (PMC10575107; doi:10.3390/s23198324)
Supplement: Supplementary file 1 [file sensors-23-08324-s001.zip › sensors-2634799-supplementary.pdf]

Table S1. The ranges of dentification parameters on different models.

Parameter	SDM/DDM		PMM	
	Lower bound	Upper bound	Lower bound	Upper bound
I_{ph} (A)	0	1	0	2
R_s (Ω)	0	0.5	0	2
R_{sh} (Ω)	0	100	0	2000
I_{sd}, I_{sd1}, I_{sd2} (μA)	0	1	0	50
n, n_1, n_2	1	2	1	50

Table S2. Parameters settings of the selected methods.

Methods	Parameters
BSA	N = 30, mix-rate=1.0
CSA	N = 30, α =0.1, β =0.15, M=3
GOTLBO	N = 30, Jr=0.8
JADE	N = 30, *Pbest* rate =0.05, c = 0.1
MPA	N = 30, *FADs* =0.2, P = 0.5
PGJAYA	N = 30
RIME	N = 30, w = 5.0
FPA	N = 30, p = 0.8
BFPA	N = 30, p = 0.95, α = 0.3

Table S3. The statistical results of different methods.

Item	Methods	Best	Median	Mean	Worst	STD
SDM	BSA	9.98587889×10^{-04}	1.26484874×10^{-03}	1.28652241×10^{-03}	1.73105320×10^{-03}	1.72×10^{-04}
	CSA	1.57400251×10^{-03}	1.88482156×10^{-03}	1.91181192×10^{-03}	2.34754518×10^{-03}	2.28×10^{-04}
	GOTLBO	9.86489640×10^{-04}	1.21098764×10^{-03}	1.22494085×10^{-03}	1.68253427×10^{-03}	1.92×10^{-04}
	JADE	9.86022997×10^{-04}	9.95295220×10^{-04}	1.04988222×10^{-03}	1.36489253×10^{-03}	1.06×10^{-04}
	MPA	1.04794349×10^{-03}	2.30772086×10^{-03}	2.38247234×10^{-03}	6.27473533×10^{-03}	1.05×10^{-03}
	PGJAYA	9.86022139×10^{-04}	9.90708089×10^{-04}	9.97068538×10^{-04}	1.04874839×10^{-03}	1.78×10^{-05}
	RIME	1.00066761×10^{-03}	1.58314674×10^{-03}	1.87409575×10^{-03}	3.80335507×10^{-03}	8.20×10^{-04}
	FPA	1.14571574×10^{-03}	1.75858880×10^{-03}	1.79679861×10^{-03}	2.39674103×10^{-03}	3.74×10^{-04}
	BFPA	**9.86021878×10^{-04}**	**9.86021878×10^{-04}**	**9.86021878×10^{-04}**	**9.86021878×10^{-04}**	**2.85×10^{-17}**
DDM	BSA	1.04455879×10^{-03}	1.40474248×10^{-03}	1.41689648×10^{-03}	2.10207873×10^{-03}	2.68×10^{-04}
	CSA	1.10028472×10^{-03}	1.26900715×10^{-03}	1.53876290×10^{-03}	2.38026591×10^{-03}	4.93×10^{-04}
	GOTLBO	1.06633593×10^{-03}	1.41450732×10^{-03}	1.39536268×10^{-03}	1.83123302×10^{-03}	2.24×10^{-04}
	JADE	9.86589096×10^{-04}	1.12071403×10^{-03}	1.29297150×10^{-03}	2.61600118×10^{-03}	4.09×10^{-04}
	MPA	1.02482502×10^{-03}	2.31374616×10^{-03}	2.27477443×10^{-03}	3.92610633×10^{-03}	7.11×10^{-04}
	PGJAYA	9.83240599×10^{-04}	9.90552971×10^{-04}	1.03246179×10^{-03}	1.33239742×10^{-03}	8.96×10^{-05}
	RIME	1.00163554×10^{-03}	2.27989212×10^{-03}	2.42612792×10^{-03}	4.40977217×10^{-03}	1.00×10^{-03}
	FPA	1.33759239×10^{-03}	2.23654730×10^{-03}	2.22696702×10^{-03}	3.36136908×10^{-03}	4.88×10^{-04}
	BFPA	**9.82484852×10^{-04}**	**9.82923813×10^{-04}**	**9.83788628×10^{-04}**	**9.86022680×10^{-04}**	**1.49×10^{-06}**
PMM	BSA	2.43257889×10^{-03}	2.45897719×10^{-03}	2.47347882×10^{-03}	2.66095159×10^{-03}	4.67×10^{-05}
	CSA	4.88686473×10^{-03}	6.21996473×10^{-03}	6.36729830×10^{-03}	8.39056856×10^{-03}	1.08×10^{-03}
	GOTLBO	2.43128427×10^{-03}	2.52563980×10^{-03}	2.53645391×10^{-03}	3.12057445×10^{-03}	1.19×10^{-04}

	JADE	2.42511242×10^{-03}	2.43177711×10^{-03}	2.44085742×10^{-03}	2.48592049×10^{-03}	1.81×10^{-05}
	MPA	2.43367927×10^{-03}	2.62402871×10^{-03}	3.18292893×10^{-03}	1.92703054×10^{-02}	3.04×10^{-03}
	PGJAYA	2.42507608×10^{-03}	2.42874533×10^{-03}	2.45231199×10^{-03}	2.64324423×10^{-03}	5.76×10^{-05}
	RIME	2.47598667×10^{-03}	2.73825329×10^{-03}	3.67252826×10^{-03}	1.11934369×10^{-02}	1.92×10^{-03}
	FPA	2.43404729×10^{-03}	2.56941663×10^{-03}	2.56811529×10^{-03}	2.62343284×10^{-03}	3.93×10^{-05}
	BFPA	**2.42507487×10^{-03}**	**2.42507487×10^{-03}**	**2.42507487×10^{-03}**	**2.42507487×10^{-03}**	**1.99×10^{-17}**

Table S4. The Wilcoxon signed rank test with α=5%.

Model	Item	Win	Tie	Lose	R^+	R^-	P Value	Hypothesis
SDM	BFPA vs BSA	30	0	0	465	0	1.73×10^{-06}	1
	BFPA vs CSA	30	0	0	465	0	1.73×10^{-06}	1
	BFPA vs GOTLBO	30	0	0	465	0	1.73×10^{-06}	1
	BFPA vs JADE	30	0	0	465	0	1.73×10^{-06}	1
	BFPA vs MPA	30	0	0	465	0	1.73×10^{-06}	1
	BFPA vs PGJAYA	30	0	0	465	0	1.73×10^{-06}	1
	BFPA vs RIME	30	0	0	465	0	1.73×10^{-06}	1
	BFPA vs FPA	30	0	0	465	0	1.73×10^{-06}	1
DDM	BFPA vs BSA	30	0	0	465	0	1.73×10^{-06}	1
	BFPA vs CSA	30	0	0	465	0	1.73×10^{-06}	1
	BFPA vs GOTLBO	30	0	0	465	0	1.73×10^{-06}	1
	BFPA vs JADE	30	0	0	465	0	1.73×10^{-06}	1
	BFPA vs MPA	30	0	0	465	0	1.73×10^{-06}	1
	BFPA vs PGJAYA	27	0	3	457	8	3.88×10^{-06}	1
	BFPA vs RIME	30	0	0	465	0	1.73×10^{-06}	1
	BFPA vs FPA	30	0	0	465	0	1.73×10^{-06}	1
PVM	BFPA vs BSA	30	0	0	465	0	1.73×10^{-06}	1
	BFPA vs CSA	30	0	0	465	0	1.73×10^{-06}	1
	BFPA vs GOTLBO	30	0	0	465	0	1.73×10^{-06}	1
	BFPA vs JADE	30	0	0	465	0	1.73×10^{-06}	1
	BFPA vs MPA	30	0	0	465	0	1.73×10^{-06}	1
	BFPA vs PGJAYA	30	0	0	465	0	1.73×10^{-06}	1
	BFPA vs RIME	30	0	0	465	0	1.73×10^{-06}	1
	BFPA vs FPA	30	0	0	465	0	1.73×10^{-06}	1

Table S5. The optimal parameter results for SDM.

Methods	I_{ph} (A)	I_{sd} (μA)	R_s (Ω)	R_{sh} (Ω)	n	RSME
BSA	0.76101083	0.33272080	0.03623004	51.99260501	1.48421452	9.98587889×10^{-04}
CSA	0.76049982	0.60990297	0.03374108	80.41166897	1.54804154	1.57400251×10^{-03}
GOTLBO	0.76079618	0.32629434	0.03632583	53.62615956	1.48220425	9.86489640×10^{-04}
JADE	0.76077429	0.32298973	0.03637701	53.71435829	1.48117442	9.86022997×10^{-04}
MPA	0.76074259	0.38846536	0.03562233	58.47357679	1.50000478	1.04794349×10^{-03}
PGJAYA	0.76077527	0.32291721	0.03637856	53.71586523	1.48115115	9.86022139×10^{-04}
RIME	0.76082520	0.34959810	0.03602140	54.55788384	1.48921854	1.00066761×10^{-03}
FPA	0.76041659	0.43246234	0.03519397	63.68218988	1.51117040	1.14571574×10^{-03}
BFPA	0.76077553	0.32302082	0.03637709	53.71852531	1.48118359	9.86021878×10^{-04}

Table S6. The error value of current and power on SDM.

Index	Observed data		Simulated current data			Simulated power data	
	V_{obs}(V)	I_{obs}(A)	I_{sim}(A)	IA_e(A)	IR_e (A)	W_{sim}(W)	WA_e(W)
1	−0.2057	0.7640	7.64087704×10^{-01}	8.77035433×10^{-05}	1.14795214×10^{-02}	$-1.57172841\times10^{-01}$	$-1.80406189\times10^{-05}$
2	−0.1291	0.7620	7.62663086×10^{-01}	6.63085899×10^{-04}	8.70191468×10^{-02}	$-9.84598044\times10^{-02}$	$-8.56043895\times10^{-05}$
3	−0.0588	0.7605	7.61355307×10^{-01}	8.55306879×10^{-04}	1.12466388×10^{-01}	$-4.47676920\times10^{-02}$	$-5.02920445\times10^{-05}$
4	0.0057	0.7605	7.60153991×10^{-01}	$-3.46009267\times10^{-04}$	$-4.54976025\times10^{-02}$	4.33287775×10^{-03}	$-1.97225282\times10^{-06}$
5	0.0646	0.7600	7.59055209×10^{-01}	$-9.44791397\times10^{-04}$	$-1.24314658\times10^{-01}$	4.90349665×10^{-02}	$-6.10335243\times10^{-05}$
6	0.1185	0.7590	7.58042345×10^{-01}	$-9.57655048\times10^{-04}$	$-1.26173261\times10^{-01}$	8.98280179×10^{-02}	$-1.13482123\times10^{-04}$
7	0.1678	0.7570	7.57091654×10^{-01}	9.16536918×10^{-05}	1.21074890×10^{-02}	1.27039979×10^{-01}	1.53794895×10^{-05}
8	0.2132	0.7570	7.56141365×10^{-01}	$-8.58635457\times10^{-04}$	$-1.13426084\times10^{-01}$	1.61209339×10^{-01}	$-1.83061079\times10^{-04}$
9	0.2545	0.7555	7.55086872×10^{-01}	$-4.13127503\times10^{-04}$	$-5.46826609\times10^{-02}$	1.92169609×10^{-01}	$-1.05140950\times10^{-04}$
10	0.2924	0.7540	7.53663878×10^{-01}	$-3.36121970\times10^{-04}$	$-4.45785106\times10^{-02}$	2.20371318×10^{-01}	$-9.82820641\times10^{-05}$
11	0.3269	0.7505	7.51390966×10^{-01}	8.90966348×10^{-04}	1.18716369×10^{-01}	2.45629707×10^{-01}	2.91256899×10^{-04}
12	0.3585	0.7465	7.47353851×10^{-01}	8.53851267×10^{-04}	1.14380612×10^{-01}	2.67926356×10^{-01}	3.06105679×10^{-04}
13	0.3873	0.7385	7.40117222×10^{-01}	1.61722183×10^{-03}	2.18987383×10^{-01}	2.86647400×10^{-01}	6.26350013×10^{-04}
14	0.4137	0.7280	7.27382225×10^{-01}	$-6.17775119\times10^{-04}$	$-8.48592197\times10^{-02}$	3.00918026×10^{-01}	$-2.55573567\times10^{-04}$
15	0.4373	0.7065	7.06972651×10^{-01}	4.72651213×10^{-04}	6.69003840×10^{-02}	3.09159140×10^{-01}	2.06690375×10^{-04}
16	0.4590	0.6755	6.75280151×10^{-01}	$-2.19848741\times10^{-04}$	$-3.25460757\times10^{-02}$	3.09953589×10^{-01}	$-1.00910572\times10^{-04}$
17	0.4784	0.6320	6.30758272×10^{-01}	$-1.24172785\times10^{-03}$	$-1.96475925\times10^{-01}$	3.01754757×10^{-01}	$-5.94042603\times10^{-04}$
18	0.4960	0.5730	5.71928358×10^{-01}	$-1.07164194\times10^{-03}$	$-1.87023027\times10^{-01}$	2.83676466×10^{-01}	$-5.31534405\times10^{-04}$
19	0.5119	0.4990	4.99607018×10^{-01}	6.07018429×10^{-04}	1.21646980×10^{-01}	2.55748833×10^{-01}	3.10732734×10^{-04}
20	0.5265	0.4130	4.13648792×10^{-01}	6.48791986×10^{-04}	1.57092490×10^{-01}	2.17786089×10^{-01}	3.41588980×10^{-04}
21	0.5398	0.3165	3.17510109×10^{-01}	1.01010942×10^{-03}	3.19149896×10^{-01}	1.71391957×10^{-01}	5.45257065×10^{-04}
22	0.5521	0.2120	2.12154939×10^{-01}	1.54939012×10^{-04}	7.30844395×10^{-02}	1.17130742×10^{-01}	8.55418283×10^{-05}
23	0.5633	0.1035	1.02251312×10^{-01}	$-1.24868828\times10^{-03}$	$-1.20646210\times10^{+00}$	5.75981639×10^{-02}	$-7.03386106\times10^{-04}$
24	0.5736	−0.0100	$-8.71754169\times10^{-03}$	1.28245831×10^{-03}	$-1.28245831\times10^{+01}$	$-5.00038192\times10^{-03}$	7.35618084×10^{-04}
25	0.5833	−0.1230	$-1.25507413\times10^{-01}$	$-2.50741270\times10^{-03}$	$2.03854691\times10^{+00}$	$-7.32084738\times10^{-02}$	$-1.46257383\times10^{-03}$
26	0.5900	−0.2100	$-2.08472326\times10^{-01}$	1.52767362×10^{-03}	$-7.27463629\times10^{-01}$	$-1.22998673\times10^{-01}$	9.01327437×10^{-04}

Table S7. The optimal parameter results for DDM.

Methods	I_{ph} (A)	I_{sd1} (μA)	R_s (Ω)	R_{sh} (Ω)	n_1	I_{sd2} (μA)	n_2	RSME
BSA	0.76054838	0.15245541	0.03694720	53.30633319	1.47197339	0.12773690	1.46127400	1.04455879×10^{-03}
CSA	0.76077567	0.63762271	0.03955983	61.25354547	1.58308689	0.00006588	1.00038212	1.10028472×10^{-03}
GOTLBO	0.76059680	0.00000000	0.03557214	59.43401052	2.00000000	0.39415479	1.50148704	1.06633593×10^{-03}
JADE	0.76072855	0.23160900	0.03652162	55.28826323	1.69272148	0.21739903	1.45396147	9.86589096×10^{-04}
MPA	0.76115553	0.14441144	0.03718630	50.15952720	1.41564064	0.86648538	1.88163953	1.02482502×10^{-03}
PGJAYA	0.76079497	0.56455684	0.03659603	55.42408081	2.00000000	0.25158810	1.46019804	9.83240599×10^{-04}
RIME	0.76112047	0.21075338	0.03682173	52.15014571	1.44510737	0.85934395	2.00000000	1.00163554×10^{-03}
FPA	0.76114221	0.41272022	0.03543291	61.48216377	1.50599519	0.00000000	1.99880982	1.33759239×10^{-03}
BFPA	0.76078108	0.22597420	0.03674043	55.48544399	1.45101674	0.74934838	2.00000000	9.82484852×10^{-04}

Table S8. The error value of current and power on DDM.

Index	Observed data		Simulated current data			Simulated power data	
	V_{obs}(V)	I_{obs}(A)	I_{sim}(A)	IA_e(A)	IR_e (A)	W_{sim}(W)	WA_e(W)
1	−0.2057	0.7640	7.63983412×10^{-01}	−1.65876719×10^{-05}	−2.17116125×10^{-03}	−1.57151388×10^{-01}	3.41208412×10^{-06}
2	−0.1291	0.7620	7.62604096×10^{-01}	6.04096031×10^{-04}	7.92776944×10^{-02}	−9.84521888×10^{-02}	−7.79887976×10^{-05}
3	−0.0588	0.7605	7.61337698×10^{-01}	8.37698239×10^{-04}	1.10150985×10^{-01}	−4.47666567×10^{-02}	−4.92566565×10^{-05}
4	0.0057	0.7605	7.60173788×10^{-01}	−3.26212207×10^{-04}	−4.28944388×10^{-02}	4.33299059×10^{-03}	−1.85940958×10^{-06}
5	0.0646	0.7600	7.59107680×10^{-01}	−8.92319936×10^{-04}	−1.17410518×10^{-01}	4.90383561×10^{-02}	−5.76438678×10^{-05}
6	0.1185	0.7590	7.58121420×10^{-01}	−8.78580368×10^{-04}	−1.15754989×10^{-01}	8.98373882×10^{-02}	−1.04111774×10^{-04}
7	0.1678	0.7570	7.57188613×10^{-01}	1.88613349×10^{-04}	2.49158981×10^{-02}	1.27056249×10^{-01}	3.16493199×10^{-05}
8	0.2132	0.7570	7.56243606×10^{-01}	−7.56393522×10^{-04}	−9.99198840×10^{-02}	1.61231137×10^{-01}	−1.61263099×10^{-04}
9	0.2545	0.7555	7.55177301×10^{-01}	−3.22698976×10^{-04}	−4.27132992×10^{-02}	1.92192623×10^{-01}	−8.21268893×10^{-05}
10	0.2924	0.7540	7.53722353×10^{-01}	−2.77646998×10^{-04}	−3.68232093×10^{-02}	2.20388416×10^{-01}	−8.11839823×10^{-05}
11	0.3269	0.7505	7.51399134×10^{-01}	8.99133688×10^{-04}	1.19804622×10^{-01}	2.45632377×10^{-01}	2.93926803×10^{-04}
12	0.3585	0.7465	7.47301443×10^{-01}	8.01442730×10^{-04}	1.07360044×10^{-01}	2.67907567×10^{-01}	2.87317219×10^{-04}
13	0.3873	0.7385	7.40010660×10^{-01}	1.51066023×10^{-03}	2.04557919×10^{-01}	2.86606129×10^{-01}	5.85078707×10^{-04}
14	0.4137	0.7280	7.27246952×10^{-01}	−7.53047816×10^{-04}	−1.03440634×10^{-01}	3.00862064×10^{-01}	−3.11535881×10^{-04}
15	0.4373	0.7065	7.06850298×10^{-01}	3.50297618×10^{-04}	4.95821116×10^{-02}	3.09105635×10^{-01}	1.53185148×10^{-04}
16	0.4590	0.6755	6.75210543×10^{-01}	−2.89457434×10^{-04}	−4.28508415×10^{-02}	3.09921639×10^{-01}	−1.32860962×10^{-04}
17	0.4784	0.6320	6.30760758×10^{-01}	−1.23924224×10^{-03}	−1.96082633×10^{-01}	3.01755947×10^{-01}	−5.92853488×10^{-04}
18	0.4960	0.5730	5.71994733×10^{-01}	−1.00526702×10^{-03}	−1.75439271×10^{-01}	2.83709388×10^{-01}	−4.98612443×10^{-04}
19	0.5119	0.4990	4.99706135×10^{-01}	7.06135253×10^{-04}	1.41510071×10^{-01}	2.55799571×10^{-01}	3.61470636×10^{-04}
20	0.5265	0.4130	4.13733673×10^{-01}	7.33672552×10^{-04}	1.77644686×10^{-01}	2.17830779×10^{-01}	3.86278599×10^{-04}
21	0.5398	0.3165	3.17546205×10^{-01}	1.04620531×10^{-03}	3.30554599×10^{-01}	1.71411442×10^{-01}	5.64741624×10^{-04}
22	0.5521	0.2120	2.12122995×10^{-01}	1.22995453×10^{-04}	5.80167231×10^{-02}	1.17113106×10^{-01}	6.79057896×10^{-05}
23	0.5633	0.1035	1.02163276×10^{-01}	−1.33672396×10^{-03}	−1.29152073×10^{+00}	5.75485734×10^{-02}	−7.52976604×10^{-04}
24	0.5736	−0.0100	−8.79175112×10^{-03}	1.20824888×10^{-03}	−1.20824888×10^{+01}	−5.04294844×10^{-03}	6.93051559×10^{-04}
25	0.5833	−0.1230	−1.25543435×10^{-01}	−2.54343465×10^{-03}	2.06783305×10^{+00}	−7.32294854×10^{-02}	−1.48358543×10^{-03}
26	0.5900	−0.2100	−2.08371588×10^{-01}	1.62841193×10^{-03}	−7.75434254×10^{-01}	−1.22939237×10^{-01}	9.60763041×10^{-04}

Table S9. The optimal parameter results for PMM.

Methods	I_{ph} (A)	I_{sd} (μA)	R_s (Ω)	R_{sh} (Ω)	n	RSME
BSA	1.03022364	3.67004181	1.19657495	1061.89575872	48.84363047	2.4325789×10^{-03}
CSA	1.03909735	0.71851380	1.34729333	377.38332989	43.26578397	4.8868647×10^{-03}
GOTLBO	1.03054734	3.60569306	1.19762664	985.64658115	48.77823538	2.4312843×10^{-03}
JADE	1.03049288	3.49859381	1.20081875	986.94251174	48.66071913	2.4251124×10^{-03}
MPA	1.03057320	3.69411165	1.19427168	1000.96281408	48.87153638	2.4336793×10^{-03}
PGJAYA	1.03050672	3.48490320	1.20119536	983.18987863	48.64571923	2.4250761×10^{-03}
RIME	1.02919421	4.15349707	1.18290035	1263.76810861	49.32607751	2.4759867×10^{-03}
FPA	1.02977599	3.64086833	1.19676648	1072.91382269	48.81370866	2.4340473×10^{-03}
BFPA	1.03051430	3.48226280	1.20127101	981.98222691	48.64283473	2.4250749×10^{-03}

Table S10. The error value of current and power on PMM.

Index	Observed data		Simulated current data			Simulated power data	
	V_{obs}(V)	I_{obs}(A)	I_{sim}(A)	IA_e(A)	IR_e (A)	W_{sim}(W)	WA_e(W)
1	0.1248	1.0315	$1.02911916\times10^{+00}$	$-2.38083837\times10^{-03}$	$-2.30813220\times10^{-01}$	1.28434071×10^{-01}	$-2.97128628\times10^{-04}$
2	1.8093	1.03	$1.02738107\times10^{+00}$	$-2.61892659\times10^{-03}$	$-2.54264718\times10^{-01}$	$1.85884058\times10^{+00}$	$-4.73842388\times10^{-03}$
3	3.3511	1.026	$1.02574180\times10^{+00}$	$-2.58202877\times10^{-04}$	$-2.51659724\times10^{-02}$	$3.43736334\times10^{+00}$	$-8.65263661\times10^{-04}$
4	4.7622	1.022	$1.02410715\times10^{+00}$	2.10715494×10^{-03}	2.06179544×10^{-01}	$4.87700309\times10^{+00}$	1.00346933×10^{-02}
5	6.0538	1.018	$1.02229180\times10^{+00}$	4.29180468×10^{-03}	4.21591815×10^{-01}	$6.18875013\times10^{+00}$	2.59817272×10^{-02}
6	7.2364	1.0155	$1.01993068\times10^{+00}$	4.43068095×10^{-03}	4.36305362×10^{-01}	$7.38062638\times10^{+00}$	3.20621797×10^{-02}
7	8.3189	1.014	$1.01636311\times10^{+00}$	2.36310572×10^{-03}	2.33047901×10^{-01}	$8.45502304\times10^{+00}$	1.96584402×10^{-02}
8	9.3097	1.01	$1.01049615\times10^{+00}$	4.96151344×10^{-04}	4.91238954×10^{-02}	$9.40741602\times10^{+00}$	4.61902016×10^{-03}
9	10.2163	1.0035	$1.00062897\times10^{+00}$	$-2.87103027\times10^{-03}$	$-2.86101671\times10^{-01}$	$1.02227257\times10^{+01}$	$-2.93313066\times10^{-02}$
10	11.0449	0.988	9.84548378×10^{-01}	$-3.45162151\times10^{-03}$	$-3.49354404\times10^{-01}$	$1.08742384\times10^{+01}$	$-3.81228144\times10^{-02}$
11	11.8018	0.963	9.59521676×10^{-01}	$-3.47832391\times10^{-03}$	$-3.61196668\times10^{-01}$	$1.13240829\times10^{+01}$	$-4.10504831\times10^{-02}$
12	12.4929	0.9255	9.22838818×10^{-01}	$-2.66118198\times10^{-03}$	$-2.87539922\times10^{-01}$	$1.15289331\times10^{+01}$	$-3.32458804\times10^{-02}$
13	13.1231	0.8725	8.72599663×10^{-01}	9.96627823×10^{-05}	1.14226685×10^{-02}	$1.14512126\times10^{+01}$	1.30788466×10^{-03}
14	13.6983	0.8075	8.07274264×10^{-01}	$-2.25736362\times10^{-04}$	$-2.79549674\times10^{-02}$	$1.10582850\times10^{+01}$	$-3.09220441\times10^{-03}$
15	14.2221	0.7265	7.28336478×10^{-01}	1.83647796×10^{-03}	2.52784302×10^{-01}	$1.03584742\times10^{+01}$	2.61185732×10^{-02}
16	14.6995	0.6345	6.37138000×10^{-01}	2.63799997×10^{-03}	4.15760437×10^{-01}	$9.36561003\times10^{+00}$	3.87772806×10^{-02}
17	15.1346	0.5345	5.36213063×10^{-01}	1.71306309×10^{-03}	3.20498239×10^{-01}	$8.11537022\times10^{+00}$	2.59265246×10^{-02}
18	15.5311	0.4275	4.29511325×10^{-01}	2.01132483×10^{-03}	4.70485339×10^{-01}	$6.67078334\times10^{+00}$	3.12380870×10^{-02}
19	15.8929	0.3185	3.18774483×10^{-01}	2.74482673×10^{-04}	8.61798030×10^{-02}	$5.06625098\times10^{+00}$	4.36232567×10^{-03}
20	16.2229	0.2085	2.07389507×10^{-01}	$-1.11049345\times10^{-03}$	$-5.32610768\times10^{-01}$	$3.36445923\times10^{+00}$	$-1.80154242\times10^{-02}$
21	16.5241	0.101	9.61671716×10^{-02}	$-4.83282836\times10^{-03}$	$-4.78497857\times10^{+00}$	$1.58907596\times10^{+00}$	$-7.98581391\times10^{-02}$
22	16.7987	−0.008	$-8.32538625\times10^{-03}$	$-3.25386249\times10^{-04}$	$4.06732812\times10^{+00}$	$-1.39855666\times10^{-01}$	$-5.46606599\times10^{-03}$
23	17.0499	−0.111	$-1.10936483\times10^{-01}$	6.35171862×10^{-05}	$-5.72226902\times10^{-02}$	$-1.89145594\times10^{+00}$	1.08296167×10^{-03}
24	17.2793	−0.209	$-2.09247266\times10^{-01}$	$-2.47265950\times10^{-04}$	1.18309067×10^{-01}	$-3.61564628\times10^{+00}$	$-4.27258253\times10^{-03}$
25	17.4885	−0.303	$-3.00863587\times10^{-01}$	2.13641311×10^{-03}	$-7.05086834\times10^{-01}$	$-5.26165284\times10^{+00}$	3.73626606×10^{-02}

Table S11. The optimal parameter optimized by BFPA at diverse irradiance under 25℃.

PV modules	Irradiance	I_{ph} (A)	I_{sd} (μA)	R_s (Ω)	R_{sh} (Ω)	n	RMSE
Multi-crystalline	200 W/m^2	1.64609182	0.00054482	0.37731977	693.13340621	1.00526877	1.41940597×10^{-03}
KC200GT	400 W/m^2	3.28764910	0.00164152	0.34879442	760.20808231	1.05974716	1.46759303×10^{-03}
	600 W/m^2	4.93425104	0.00428176	0.33468345	757.59233988	1.10946146	1.42605190×10^{-03}
	800 W/m^2	6.56829338	0.00171108	0.34725645	971.98166206	1.06257598	3.88076100×10^{-03}
	1000 W/m^2	8.21502928	0.00289278	0.34006651	895.87425192	1.08890343	2.45314979×10^{-03}
Mono-crystalline	200 W/m^2	0.69150980	0.14641535	0.28660654	448.21114321	1.38066251	3.20694650×10^{-04}
SM55	400 W/m^2	1.38284436	0.10041531	0.39666425	427.04853066	1.35198509	7.07550905×10^{-04}
	600 W/m^2	2.07089696	0.15548337	0.33051596	450.05498502	1.38751772	8.23694730×10^{-04}
	800 W/m^2	2.76038170	0.14395762	0.33758505	459.88014836	1.38114824	6.68957647×10^{-04}
	1000 W/m^2	3.45010312	0.17117382	0.32914250	483.91634763	1.39576242	1.14593887×10^{-03}

Thin-film ST40	200 W/m^2	0.53313748	1.42961336	1.18575100	344.98274457	1.74710283	4.77207029×10^{-04}
	400 W/m^2	1.06754397	1.84883695	1.08056985	362.51801446	1.77853676	6.30835834×10^{-04}
	600 W/m^2	1.60480945	1.44191024	1.11260797	347.69711511	1.74512745	6.74028226×10^{-04}
	800 W/m^2	2.13801481	1.15810495	1.12528628	332.88869608	1.71867399	7.74145986×10^{-04}
	1000 W/m^2	2.67579975	1.52880486	1.11322513	357.59787072	1.75032678	7.34207806×10^{-04}

Table S12. The optimal parameter optimized by BFPA at different temperatures under 1000 W/m^2.

PV modules	Temperature	I_{ph} (A)	I_{sd} (μA)	R_s (Ω)	R_{sh} (Ω)	n	RMSE
Multi-crystalline	25℃	8.21440098	0.00305062	0.33875797	924.92112398	1.09150236	2.75958568×10^{-03}
KC200GT	50℃	8.29530599	0.12593260	0.33565856	953.79513259	1.11728235	2.74632793×10^{-03}
	75℃	8.37766334	1.63086359	0.34249674	790.54609087	1.10148163	4.47064521×10^{-03}
Mono-crystalline	25℃	4.93408935	0.00411685	0.33577319	756.03951527	1.10739010	1.34480754×10^{-03}
SM55	40℃	3.28662231	0.00194388	0.34281529	813.02399445	1.01694478	1.62874637×10^{-03}
	60℃	1.64616713	0.00057178	0.37289300	691.38759612	1.35243208	1.42314651×10^{-03}
Thin-film ST40	25℃	2.67579975	1.52880479	1.11322513	357.59786915	1.75032677	7.34207806×10^{-04}
	40℃	2.68091215	5.66605502	1.12929801	364.10670651	1.72255682	1.32166203×10^{-03}
	55℃	2.69196884	18.67997841	1.14959391	295.01766312	1.71757078	1.82375586×10^{-03}
	70℃	2.69232889	87.52623900	1.12588313	367.76428759	1.72732815	7.77862370×10^{-04}
